# Assessment of the ecological bias of seven aggregate social deprivation indices

**DOI:** 10.1186/s12889-016-4007-8

**Published:** 2017-01-17

**Authors:** Josephine Bryere, Carole Pornet, Nane Copin, Ludivine Launay, Gaëlle Gusto, Pascale Grosclaude, Cyrille Delpierre, Thierry Lang, Olivier Lantieri, Olivier Dejardin, Guy Launoy

**Affiliations:** 1 “Cancers & Préventions” U1086 INSERM-UCN, Centre François Baclesse, Avenue Général Harris, 14076 Caen, France; 2 Pôle recherche, CHU de Caen, Avenue de la Côte de Nacre, 14033 Caen, France; 3 Université de Caen Normandie, Esplanade de la paix, 14000 Caen, France; 4 IRSA, 45 rue de la Parmentière, 37521 La Riche, Cedex France; 5 Centre François Baclesse, Avenue Général Harris, 14076 Caen, France; 6 UMR 1027 INSERM, Faculté de médecine, 37 allée Jules Guesde, 31073 Toulouse, France; 7 Université Toulouse III - Paul Sabatier, 118 route de Narbonne, 31062 Toulouse, cedex 9 France

**Keywords:** Ecological social deprivation indices, Social inequalities in health, Aggregate-level, Individual-level

## Abstract

**Background:**

In aggregate studies, ecological indices are used to study the influence of socioeconomic status on health. Their main limitation is ecological bias. This study assesses the misclassification of individual socioeconomic status in seven ecological indices.

**Methods:**

Individual socioeconomic data for a random sample of 10,000 persons came from periodic health examinations conducted in 2006 in 11 French departments. Geographical data came from the 2007 census at the lowest geographical level available in France. The Receiver Operating Characteristics (ROC) curves, the areas under the curves (AUC) for each individual variable, and the distribution of deprived and non-deprived persons in quintiles of each aggregate score were analyzed.

**Results:**

The aggregate indices studied are quite good “proxies” for individual deprivation (AUC close to 0.7), and they have similar performance. The indices are more efficient at measuring individual income than education or occupational category and are suitable for measuring of deprivation but not affluence.

**Conclusions:**

The study inventoried the aggregate indices available in France and evaluated their assessment of individual SES.

## Background

Evidence-based policy-making for reducing social disparities in health requires measuring disparities accurately and to follow trends over time. Various approaches are used to measure socioeconomic status (SES). At the individual level, SES is mainly explored in three domains: income, education and occupational status [1]. At an aggregate level, publicly available measures of SES in residential areas are frequently used [2]. Several geographical composite indices have been created, these are known as ecological deprivation indices. As described by Townsend, deprivation, a “state of observable and demonstrable disadvantage relative to the local community or the wider society to which an individual, family or group belongs”, is a broad multidimensional concept that is closely linked to poverty. “The concept of deprivation covers the various conditions independent of income, experienced by people who are poor” [3]. Evaluate deprivation in its entire dimension suggests that the proper evaluation of the social environment should not be limited to any particular indicator such as financial resources, education or profession. Geographical approaches are thus particularly relevant for studying social inequalities in health. Measuring only one of the components of deprivation is insufficient to correctly classify communities [4], while deprivation indices, by their composite nature, are less sensitive to measurement bias and provide a comprehensive approach to deprivation [5, 6].

Deprivation indices, which are mainly derived from population census data, were first developed in the 1970s in the United Kingdom, the United States and Canada [3, 4, 7–12]. They have been implemented more recently in Europe [13–20]. Their main limitation when used to approximate individual SES is ecological bias, leading to misclassification. Ecological bias is a particular bias related to studies using aggregate data. It can lead to estimation error of the degree of association between exposure and effect. Individuals who have had an effect are not necessary those who were exposed. One way to minimize ecological bias is to use the lowest geographical unit [21], although even at the lowest geographical level, ecological bias is expected to persist.

The overall objective of this study was to assess the ecological bias induced by using seven deprivation indices that evaluate deprivation at the lowest geographical unit level for which census data are available in France: Townsend index [3], Carstairs index [8], Lasbeur index [14], Havard index [15], European Deprivation index (EDI) [18], and the social (SCP) and material (MCP) components of Pampalon index [12, 22].

## Methods

A general population sample was constituted in northwest France using exact known addresses allowing geolocalization and geocoding for IRIS (Ilots Regroupés pour l’Information Statistique). An IRIS comprises an average of 2000 inhabitants as defined by INSEE (National Institute of Statistics and Economic Studies). On 1st January 2009, Metropolitan France contained 16,076 IRIS based on divisions of urban municipalities of at least 10,000 inhabitants. Most municipalities had 5000 to 10,000 inhabitants. For the smallest undivided municipalities (34,115), an IRIS is considered equivalent to the municipality [23].

For each deprivation index, ecological bias was assessed by comparing the deprivation level of the IRIS with the individual level socioeconomic characteristics.

### Study population

Approximately 85% of the French population affiliated with the general health coverage system is invited to a periodic health examination in a health examination center (HEC). The study sample of 10,000 subjects consisted of individuals 16 years and older who consulted in 2006 at one of the 11 HEC located in northwest France—about 60,000 people. The study sample average age was 44.34 years (median 45 years), older than the French general population average age of 37.9 years (median 37.9). Compared with the French general population rates, the rate of Couverture Maladie Universelle (CMU), rate of unemployment among the active population, and rate of people without diploma were 10.3 vs 3.4%, 8.4 vs 8.8%, and 14.2 vs 19.4%, respectively [24]. The CMU is a French public health welfare program. For people with low incomes (less than 720€ per month), the CMU offers complementary 100% health coverage, which is added to standard Social Security payments; this avoids the necessity of additional private insurance.

Since the geocoding process was not fully automated and was relatively intensive, a random sample of 10,000 people was used. Among these, 402 could not be geocoded because they lived in a neighboring department.

### Individual data

All subjects were interviewed about four characteristics:

- Their education level.
- Their occupation and position.
- The feeling of having financial difficulties, as assessed by the following question “Are there times of the month when you are having real financial problems in meeting your needs (food, rent, electricity)?”
- If they receive CMU.

### Calculation of the seven aggregated deprivation indices

Two British indices, Townsend and Carstairs, were calculated based on the unweighted sum of four socioeconomic standardized variables.

SCP, MCP (designed in Canada) were calculated using principal component analysis (PCA) [12–16, 19]. Unlike the components of other indices built by PCA, SCP and MCP components were selected a priori according to the literature [12, 16].

The Havard and Lasbeur scores were designed in France and were defined as the first principal components of a PCA of nine (Havard) or 19 (Lasbeur) 2007 census variables [14, 16]. The SCP and MCP were calculated from the first two principal components of a six-variable PCA [12, 22].

The methodology of the French EDI [18] is based on the weighted combination of geographical census variables correlated with an individual indicator of deprivation, itself obtained from individual data from the European Union Statistics on Income and Living Conditions (EU-SILC) survey.

The aggregate socioeconomic data in IRIS were obtained from the 2007 census for homogeneity with the individual data from 2006. A version in national quintiles was available for each deprivation index.

### Statistical analysis

Each individual-level socioeconomic variable was dichotomized as follows. “Education level”: having a diploma/not having a diploma. “Occupation and position”: employed/unemployed. “Financial difficulties”: having/not having financial difficulties. “CMU”: yes/no. People were synthetically considered deprived at the individual level if they were disadvantaged in at least two of the four variables presented above. We set a threshold of at least two variables both because we wanted to ensure that at least two dimensions of deprivation were integrated and because with a threshold of at least three variables, less than 4% of the population would have been considered deprived.

Ecological bias was first assessed by the Receiver Operating Characteristics (ROC) curves of each deprivation index according to each individual variable. The ROC curve plots the true positive rate (sensitivity) as a function of the false positive rate (100-specificity) for different deprivation index cut-off points. Each point of the ROC curve represents a sensitivity/specificity pair corresponding to a particular decision threshold. In our study, the ROC curves are used to understand how the indices can appreciate individual deprivation. We also calculated the Area Under the Curves (AUC), defined as the measure of how well the indices can distinguish between the deprived and the non-deprived. An AUC close to 1 means that the aggregate index perfectly distinguishes individual deprivation, while an AUC close to 0.5 means that the aggregate index does not distinguish individual deprivation better than chance. An AUC between 0.9 and 1 means that the index is excellent, an AUC between 0.8 and 0.9 means that the index is good, an AUC between 0.7 and 0.8 means that the index is fair, an AUC between 0.6 and 0.7 means that the index is poor and an AUC between 0.5 and 0.6 means that the index is bad.

In a complimentary approach, we analyzed the ecological bias representing the distribution of people considered deprived (or not deprived) at an individual level according to the quintile version of the different aggregate indices.

All statistical analyses were performed using SAS systems software (Statistical Analysis System software version 9.3, Cary, NC, USA).

## Results

### ROC curves (Fig. 1)

ROC Curves were constructed to evaluate the sensitivity and specificity of all seven aggregate indices, using individual deprivation as defined above as the gold standard. This analysis showed that no aggregate index was highly performant at discriminating between favored and deprived subjects at an individual level. The MCP has a ROC curve that is closer to the diagonal, suggesting that it is less adapted to capture individual deprivation. The six other ecological deprivation indices have similar performance levels. Between the point with a sensitivity of 50% and specificity of 75% and the point with a sensitivity of 75% and a specificity of 50%, Havard, Townsend and EDI seem more performant.

**Fig. 1:**
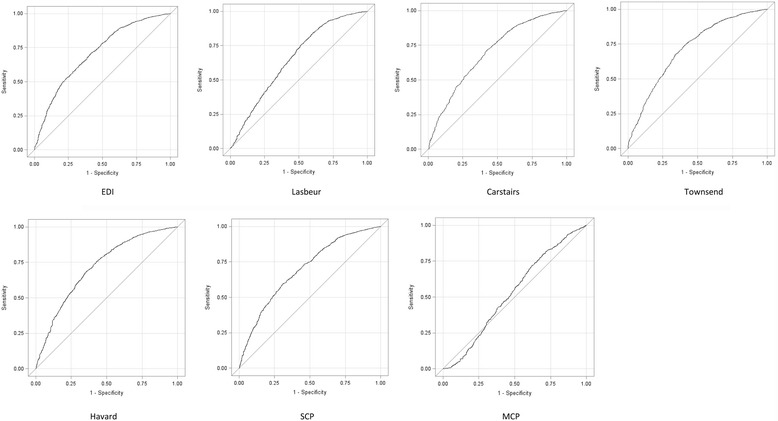
ROC Curves of different ecological indices according to individual deprivation (aggregate data from population census in 2007, individual data from IRSA 2006, *N* = 9598)

### AUC of each aggregate deprivation index according to each individual variable (Table 1)

**Table 1:** Areas under curves (AOC) and 95% CI of the receiver operating characteristics of ecological deprivation indices according to individual variables (aggregate data from population census in 2007, individual data from IRSA 2006, *N* = 9598)

Individual variables	Financial difficulties	Education	Occupation	CMU	Individual deprivation
EDI	0.647 [0.632; 0.662]	0.595 [0.578; 0.612]	0.645 [0.625; 0.666]	0.700 [0.682; 0.719]	0.708 [0.691; 0.726]
Lasbeur	0.615 [0.600; 0.631]	0.612 [0.5950; 0.628]	0.597 [0.575; 0.619]	0.643 [0.623; 0.663]	0.652 [0.633; 0.672]
Carstairs	0.643 [0.628; 0.657]	0.603 [0.587; 0.620]	0.630 [0.609; 0.651]	0.693 [0.675; 0.712]	0.696 [0.678; 0.714]
Townsend	0.650 [0.635; 0.665]	0.581 [0.5640; 0.598]	0.648 [0.627; 0.669]	0.712 [0.695; 0.731]	0.716 [0.699; 0.734]
Havard	0.644 [0.629; 0.660]	0.567 [0.549; 0.585]	0.653 [0.631; 0.675]	0.711 [0.692; 0.730]	0.717 [0.699; 0.735]
SCP	0.633 [0.618; 0.649]	0.585 [0.566; 0.603]	0.631 [0.610; 0.651]	0.687 [0.669; 0.706]	0.695 [0.677; 0.713]
MCP	0.510 [0.493; 0.527]	0.545 [0.527; 0.564]	0.546 [0.524; 0.569]	0.534 [0.512; 0.556]	0.529 [0.507; 0.550]

In accordance with the ROC curves, the AUC values and their confidence intervals indicate that the Lasbeur index and the MCP show significantly weaker performance than other indices. The MCP provided a particularly poor assessment of individual deprivation, with a value of AUC (0.529), close to the 0.5 value that is equivalent to chance. Other indices have AUC values of close to 0.7, which classifies them as “fair”.

AUC values for “CMU” were very close to those for individual deprivation. Individual low education was very poorly identified, with a very low AUC value. The AUC values for “financial difficulties” and “low occupation class” were intermediate. The EDI, Townsend and Havard indices achieved the best values except for in education, where the Lasbeur and Carstairs indices had higher AUC values. The EDI, Townsend and Havard indices were the best proxies for variables related to income, while Lasbeur and Carstairs were the best proxies for variables related to education.

### Distribution of deprived and non-deprived people (according to the individual variable) into the different quintiles of the ecological deprivation indices (Tables 2 and 3)

More than 50% of disadvantaged individuals lived in an IRIS of quintile 5 for the Townsend and Havard indices. Between 40 and 50% of them lived in an IRIS of quintile 5 for EDI, Carstairs and SCP. Less than 40% of them lived in an IRIS of quintile 5 for Lasbeur and MCP. Quintiles 4 and 5 of EDI, Carstairs, Townsend, Havard and SCP captured more than 65% of the deprived population (more than 70% for Townsend and Havard). Less than 10% of disadvantaged people live in the richest category for EDI, Carstairs, Townsend, Havard and SCP. For EDI, Carstairs, Townsend and SCP, the higher the aggregate social category, the less it contains people who are disadvantaged at the individual level (Table 2).

Analyzing the distribution of non-disadvantaged individuals showed that they were divided roughly equally into categories. The indices capture deprivation and not affluence (Table 3).

## Discussion

Ecological bias is unavoidable when assessing deprivation using aggregate indices, even when small geographical units are used. Using different approaches, our results show that none of the seven deprivation indices is clearly better than the others. Index performances are not substantially different, except for MCT, which showed particularly low performance.

This study has some methodological limitations. First, the measure of individual deprivation that was used as our “gold standard” is not a validated index. It was built using only four components (education, employment, financial difficulties and CMU). However, it had the advantage of being available in a large general population sample and of using variables known to best reflect deprivation both at the individual level and at the ecological level. We considered an individual to be deprived according to the rationale described in the Methods section. We could have chosen another threshold, but our goal was to integrate various aspects of social deprivation and to capture a proportion of the population that could be reasonably considered disadvantaged. Second, income was not directly measured at the individual level. As measures, we used both a subjective question on financial difficulties and whether the individual was covered by CMU, which is offered if the income is below 720€ for a single person. This variable objectively measures the level of income.

Selection bias is also not excluded in our sample. The study participants were not representative of the general population in age, rate of CMU coverage, and rate of people without a diploma. This is probably true of other variables. The individuals in the study sample volunteered for a periodic health examination that primarily targets people in a precarious situation, which could explain the non-representativeness of the sample population. Moreover, non-geocoded people were older and more often male than geocoded people. However, were this bias to exist, it would have little impact on the results. Non-geocoded people accounted for only 4% of the study population. The mean of each deprivation index and its distribution in the study population were very close to those in the general population.

**Table 2:** Distribution of deprived people (*N* = 1005) into the different quintiles of the ecological deprivation indices according to the individual variable

Index	Category	Percentage
EDI	Quintile 1	7.66%
	Quintile 2	9.15%
	Quintile 3	16.22%
	Quintile 4	23.58%
	Quintile 5	43.38%
Lasbeur	Quintile 1	14.13%
	Quintile 2	14.93%
	Quintile 3	15.82%
	Quintile 4	18.91%
	Quintile 5	36.22%
Carstairs	Quintile 1	4.98%
	Quintile 2	11.14%
	Quintile 3	17.61%
	Quintile 4	23.48%
	Quintile 5	42.79%
Townsend	Quintile 1	5.17%
	Quintile 2	9.15%
	Quintile 3	11.14%
	Quintile 4	23.58%
	Quintile 5	50.95%
Havard	Quintile 1	8.85%
	Quintile 2	7.36%
	Quintile 3	10.02%
	Quintile 4	22.28%
	Quintile 5	51.49%
SCP	Quintile 1	7.64%
	Quintile 2	9.34%
	Quintile 3	14.01%
	Quintile 4	21.76%
	Quintile 5	47.24%
MCP	Quintile 1	21.55%
	Quintile 2	14.86%
	Quintile 3	16.35%
	Quintile 4	15.39%
	Quintile 5	31.85%

**Table 3:** Distribution of non-deprived people (*N* = 8593) into the different quintiles of the ecological deprivation indices according to the individual variable

Index	Category	Percentage
EDI	Quintile 1	22.47%
	Quintile 2	20.60%
	Quintile 3	19.24%
	Quintile 4	21.76%
	Quintile 5	15.93%
Lasbeur	Quintile 1	23.32%
	Quintile 2	22.39%
	Quintile 3	21.13%
	Quintile 4	20.00%
	Quintile 5	13.15%
Carstairs	Quintile 1	15.71%
	Quintile 2	21.29%
	Quintile 3	24.12%
	Quintile 4	23.96%
	Quintile 5	15.52%
Townsend	Quintile 1	15.57%
	Quintile 2	20.60%
	Quintile 3	17.57%
	Quintile 4	25.99%
	Quintile 5	20.27%
Havard	Quintile 1	24.42%
	Quintile 2	16.21%
	Quintile 3	14.60%
	Quintile 4	24.45%
	Quintile 5	20.32%
SCP	Quintile 1	22.22%
	Quintile 2	18.74%
	Quintile 3	18.32%
	Quintile 4	18.46%
	Quintile 5	22.27%
MCP	Quintile 1	18.31%
	Quintile 2	20.54%
	Quintile 3	19.89%
	Quintile 4	18.89%
	Quintile 5	22.37%

The individual deprivation variable built in this study includes variables that are both objective (education level, profession, access to free medical care) and subjective (the feeling of having financial difficulties). The relatively good performance of Townsend and EDI for this multidimensional score is not surprising because both are based on a common theoretical concept: the individual experience of multidimensional deprivation [3, 18]. Other similarities in the performance of indices could be explained by their methodology and resulting composition, such as Carstairs and EDI regarding unidimensional deprivation. Indeed, EDI is composed of variables included in Townsend and Carstairs. In general, the indices are more efficient at measuring individual income than education or occupational category, and they are only suitable for measuring deprivation and not affluence.

Townsend and Carstairs indices are based on the sum of four variables: crowded households, households with no-car, percentage of unemployed and dwellings occupied by non-owners for Townsend and unskilled workers for Carstairs. Carstairs includes a dimension of education in its calculation which may explain its ability to better assess individual education. Morevover, education also occupies a more important place in the calculation of the Lasbeur index through the variables used (percentage of workers, of managers, of persons with a primary level of study) which may explain its best performance to measure individual education. However, EDI, Townsend and Havard indices are mainly composed of variables reflecting income (percentage of non-owners, households without cars, the number of unemployed…) which may explain their better ability to assess individual income.

One way to improve the performance of deprivation indices is to redefine the boundaries of the geographic areas from which the indices are constructed. The administrative boundaries of these geographic areas do not necessarily coincide with neighborhood boundaries as perceived by people. However, it appears impossible to avoid using administrative boundaries because they are the only way to use census data. Lalloué proposed creating socioeconomic categories instead of defining deprivation quintiles using hierarchical clustering that provides categories with more homogeneous compositions [19].

We chose the IRIS level because it was the smallest geographic area available in France for which we know the census data needed to calculate these indices. Moreover, it has been shown that to reduce the ecological bias, it is essential to choose the smallest geographical unit available [25, 26]. Because our goal was to determine which indices had the lowest ecological bias, in other words the ones that are closest to the individual deprivation, we restricted our study at the IRIS level and had not extend it at a broader level like municipalities.

This paper was designed to evaluate the extent that ecological deprivation indices can be considered good “proxies” of individual SES. The relevance of ecological indices is clearly not confined to this role, because they also integrate the potential effect of areas themselves. Regardless of the health event being studied—for example, disease occurrence, disease management or disease lethality—a deprived area can influence health events through the higher proportion of disadvantaged individuals in these areas (composition effect), or through aspects specific to the area (positive or negative externalities) associated with disease risk and disease management (context effect).

For example, for lung cancer disease occurrence, context effects suggest that the social structure of the area of residence influences the percentage of smokers [27]. Nearby shops in deprived areas are more densely populated; this increases the proportion of smokers [28], and the deprived areas are more polluted [29]. Consequently, ecological deprivation indices could be analyzed in multilevel statistical models as a contextual measure of SES, characterizing the SES of a neighborhood with elements of the collective composition of the territory rather than as proxies of individual SES. The results of a recent British study support this conclusion by showing the separate effects on morbidity of individual and neighborhood deprivation as measured by the English Index of Multiple Deprivation (IMD) [30].

Regarding disease management and lethality (for example for cancer), contextual effects are even better documented. Geographical and social distance from health service providers is clearly implicated as a possible explanation in increasing numbers of papers. Even if aggregate deprivation indices are precious tools to explore social inequities in health, it will be useful to have multivariate analysis aggregated indices at our disposal that are built at the same geographic scale that allow researchers to precisely assess the health isolation of these geographic entities.

The multilevel studies also seem more relevant than studies based only on individual data because they may induce an atomistic fallacy that occurs by drawing inferences regarding variability across groups. It arises because associations between two variables at the individual level may differ from associations between analogous variables measured at the group level [31]. As concluded by Salmond and Crampton, for maximum effectiveness, targeting of health resources and interventions requires a mix of area based and individual approaches [32]. The interest of using ecological indices is then to take into account the variability across groups.

## Conclusion

Even if ecological bias is unavoidable, it remains important to measure its magnitude to provide the elements for epidemiologist to measure quality of theirs studies because ecological indices are still a useful tool to evaluate social inequalities in health.

## Abbreviations

CMU: Universal health coverage
EDI: European deprivation index
HEC: Health examination center
INSEE: National Institute of Statistics and Economic Studies
IRIS: Areas grouped for statistical information
MPC: Material component of Pampalon index
SCP: Social component of Pampalon index
SES: Socioeconomic status
